# Application of a partial cell recycling chemostat for continuous production of aroma compounds at near-zero growth rates

**DOI:** 10.1186/s13104-019-4213-4

**Published:** 2019-03-25

**Authors:** Oscar van Mastrigt, Reinier A. Egas, Søren K. Lillevang, Tjakko Abee, Eddy J. Smid

**Affiliations:** 1 Food Microbiology, Wageningen University & Research, Wageningen, The Netherlands; 2 Arla Innovation Centre, Arla Foods Amba, Aarhus N, Denmark

**Keywords:** Lactic acid bacteria, Fermentation, Metabolomics, Continuous cultivation, Retentostat, Maintenance, VOC

## Abstract

**Objective:**

The partial cell recycling chemostat is a modification of the chemostat in which cells are partially recycled towards the bioreactor. This allows using dilution rates higher than the maximum growth rate resulting in higher biomass concentrations and increased process rates. In this study, we demonstrate with a single observation that this system can also be used to study microorganisms at near-zero growth rates and as production system for compounds specific for slow growth, such as those typical for ripened cheese.

**Results:**

*Lactococcus lactis* FM03-V2 was cultivated at growth rates between 0.0025 and 0.025 h^−1^. Detailed analysis of produced aroma compounds revealed that levels of particular compounds were clearly affected by the growth rate within the studied range demonstrating that we can steer the aroma production by controlling the growth rate. With this approach, we also experimentally validated that the maintenance coefficient of this dairy strain decreased at near-zero growth rates (6.4-fold). An exponentially decreasing maintenance coefficient was included in the growth model, enabling accurate prediction of biomass accumulation in the partial cell recycling chemostat. This study demonstrates the potential of partial cell recycling chemostat both as aroma production system at near-zero growth rates and as unique research tool.

**Electronic supplementary material:**

The online version of this article (10.1186/s13104-019-4213-4) contains supplementary material, which is available to authorized users.

## Introduction

The partial cell recycling chemostat (PCRC) is a modification of the chemostat in which biomass is partially recycled by means of centrifugation, filtration or gravity sedimentation [1]. Although this technique has mainly been applied to obtain higher biomass concentrations and increased process rates [2], it could also be used to grow microorganisms at extremely low growth rates in steady states. By controlling the dilution rate (D) and the recycle ratio (R), i.e. ratio of flow of cell-free permeate over the total effluent flow, the growth rate (µ) can be controlled (see Eq. 1).

1 $$\mu = \left( {1 - R} \right)\cdot{\text{D}}$$

The effect of near-zero growth rates on the physiology of microbes has been studied using retentostat cultivation (i.e. with complete cell recycling; R = 1), which never leads to a steady state [3–8]. Moreover, the growth rate quickly decreases to < 0.005 h^−1^ in retentostat cultures, making it impossible to properly study growth rates between approximately 0.005 and 0.025 h^−1^ using retentostat cultures. Recently, dynamic modelling of retentostat cultures revealed a sevenfold reduction in the maintenance requirement of *Lactococcus lactis* FM03-V1 after 35 days of cultivation [8]. Moreover, formation of particular aroma compounds increased during the cultivations resembling aroma formation during cheese ripening.

Based on this we hypothesize that the PCRC technique could support two novel applications: (i) studying microbes at near-zero growth rates in steady states and (ii) production of metabolites typical for slow growth, such as aroma compounds formed during cheese ripening. Therefore, the dairy *L. lactis* FM03-V2 was grown in a single PCRC culture at low growth rates (0.0025–0.025 h^−1^) to quantify the maintenance requirement and to assess the complexity of aroma formation at near-zero growth rates. Moreover, additional applications of the PCRC are discussed.

## Main text

### Methods

#### Strain and medium

We used *Lactococcus lactis* subsp. *lactis* FM03-V2, which is a single colony isolate of *L. lactis* FM03-V1 lacking plasmid pLd1 carrying the *citQRP* operon [8]. For the continuous cultivation, a single colony from a LM17 agar plate containing 0.5% (w/v) lactose was cultured overnight at 30 °C on lactose-limited chemically defined medium [9]. This culture was used to inoculate the bioreactor at 1% (v/v). For the continuous cultivation a chemically-defined medium was used containing 0.5% (w/w) lactose, 10 mM (NH_4_)_3_ citrate and 1% Bacto-tryptone [9].

#### Partial cell recycling chemostat cultivation

Bacteria were grown in a partial cell recycling chemostat (PCRC) culture at growth rates between 0.0025 and 0.025 h^−1^ in a bioreactor with a working volume of 1 L (Multifors, Infors HT, Switzerland; see Additional file 2: Figure S1 for a schematic representation). A constant dilution rate of 0.025 h^−1^ was used and the growth rate was varied by changing the recycle ratio by removing part of the effluent via a polyethersulfone cross-flow filter (0.65 µm, Spectrum laboratories, USA). The temperature was kept constant at 30 °C, the pH was controlled at 5.5 by automatic addition of 5 M NaOH and anaerobic conditions were maintained by flushing the headspace with nitrogen gas at 0.1 L min^−1^. The stirring speed was set at 400 rpm. The optical density at 600 nm was continuously monitored using an internal probe (Trucell2, Finesse, USA). Samples were taken after reaching steady-state conditions, which were considered to be achieved when the online optical density did not change significantly for approximately 2 days. For the lowest growth rate, samples were taken after 6 volume changes. Rates of the feed, effluent and cell-free permeate were determined by continuously weighing the medium and waste vessels.

#### Biomass determination

The cell dry weight was determined in triplicate as previously described [9]

#### Cell viability

The viability of cells in the culture was determined in every steady state by LIVE/DEAD *Bac*light Bacterial Viability kit (Molecular Probes Europe, The Netherlands) as previously described [8]. At least 2 pictures per steady state with at least 200 cells per picture were counted.

#### Extracellular metabolites analysis

Lactose, citrate, lactate, acetate, ethanol, formate, pyruvate and acetoin were quantified with technical duplicates by high performance liquid chromatography (HPLC) as previously described [9].

#### Volatile organic compounds analysis

Volatile organic compounds were analyzed with technical duplicates by headspace solid phase microextraction gas chromatography mass spectrometry (HS SPME GC–MS) as previously described [8].

#### Modeling

To estimate the energy-related maintenance coefficient (m_ATP_) in time, biomass accumulation during the PCRC cultivation was fitted using Eq. 2 (see Additional file 1) assuming a constant maximum biomass yield on ATP (Y_x/ATP_^max^) of 15.94 gDW/mol ATP [8].

2 $$C_{X} \left( t \right) = \left( {C_{x,0} - \frac{{\left( {\frac{F}{V} \cdot \left( {C_{s,in} - C_{s} } \right) \cdot Y_{x/ATP}^{max} \cdot Y_{ATP/s} } \right)}}{{Y_{x/ATP}^{max} \cdot m_{ATP} - \left( {1 - R} \right) \cdot \frac{F}{V}}}} \right) \cdot e^{{ - \left( {Y_{x/ATP}^{max} \cdot m_{ATP} + \frac{F}{V} \cdot \left( {1 - R} \right)} \right) \cdot t}} + \frac{{\left( {\frac{F}{V} \cdot \left( {C_{s,in} - C_{s} } \right) \cdot Y_{x/ATP}^{max} \cdot Y_{ATP/s} } \right)}}{{Y_{x/ATP}^{max} \cdot m_{ATP} - \left( {1 - R} \right) \cdot \frac{F}{V}}}$$

The ATP yield on substrate (Y_ATP/s_) was estimated based on the measured metabolite production:

3 $$Y_{ATP/S} = \frac{{R_{lactate} + R_{ethanol} + R_{pyruvate} + 2 \cdot R_{acetoin} + 2 \cdot R_{acetate} }}{{ - \left( {12 \cdot R_{lactose} } \right)}}$$

in which R_i_ is the production rate (mol/h) of compound i.

Input data for the fitting were the online optical density measurements, which were first converted into biomass dry weight concentrations using a second-order polynomial relation (Additional file 2: Figure S2). During fitting, the sum of squared errors between the model and the input data was minimized in 10-min intervals using the solver add-in of Excel by estimating the m_ATP_, which was assumed to gradually decrease at near-zero growth rates [8]:

4 $$m_{ATP} = m_{ATP,che} - a \cdot e^{ - b \cdot \mu }$$

in which m_ATP.che_ denotes the m_ATP_ as found by extrapolation from chemostat cultures (2.43 mmol ATP gDW^−1^ h^−1^) and a and b are the fitted parameters.

### Results

**Fig. 1:**
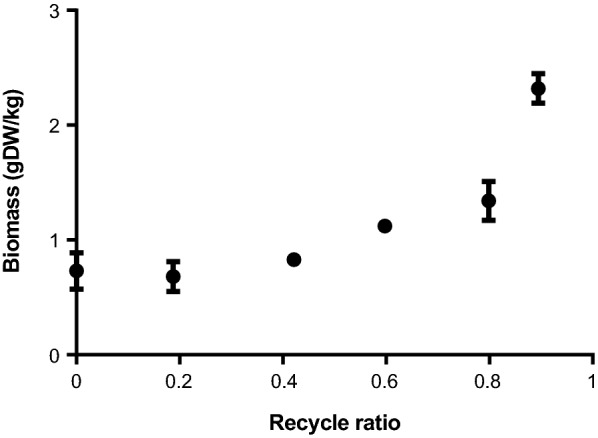
Biomass concentration in steady states of a partial cell recycling chemostat culture at different recycle ratios. Error bars represent the standard deviation of technical triplicates

*Lactococcus lactis* FM03-V2 was grown in a partial cell recycling chemostat (PCRC) bioreactor with a constant dilution rate (0.025 h^−1^) at various recycling ratios (0 to 0.9) resulting in growth rates between 0.025 and 0.0025 h^−1^. The biomass concentration increased 3.4-fold at higher recycle ratios (Fig. 1). Throughout the cultivation, the viability remained high (> 99%) as determined by live/dead staining.

#### Metabolism

The main carbon source (lactose) and the main fermentation products (lactate, acetate, ethanol, formate, acetoin and pyruvate) were quantified in all steady states at growth rates between 0.025 and 0.0025 h^−1^ using HPLC (Additional file 2: Figure S3). Residual lactose concentrations were below the detection limit suggesting lactose-limited conditions. The lactose was converted mainly into lactate, acetate, formate, ethanol and pyruvate. At lower growth rates, lactate production slightly increased (from 53 to 63%), while the formation of acetate, ethanol and formate decreased (from 47 to 36%). Production of formate was only 60% of the sum of acetate and ethanol indicating that pyruvate was converted into acetyl-CoA via both pyruvate formate lyase (PFL) and pyruvate dehydrogenase (PDH) (Additional file 2: Figure S3).

#### Maintenance estimation

**Fig. 2:**
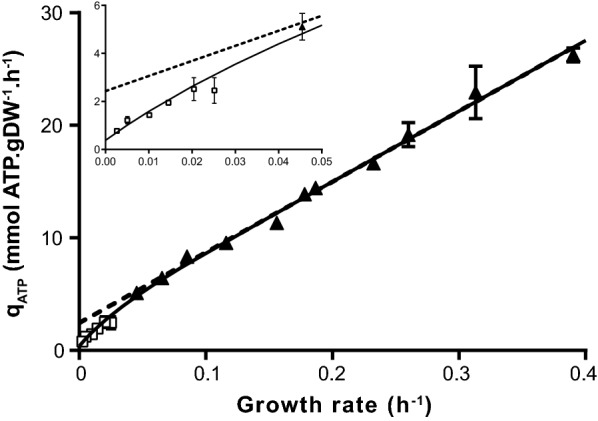
Relation between biomass specific ATP production rate (q_ATP_) and the growth rate. The filled triangles and squares represent measurements from chemostat cultures and a partial cell recycling chemostat cultures, respectively. The dashed line is a linear regression line based on data from chemostat cultures which is used to estimate the maximum biomass yield on ATP (− 1/slope) and the maintenance coefficient (intercept). The black line represent the model in which the maintenance exponentially decreases at low growth rates (Eq. 4). The axis of the inset are the same as of the main figure. Error bars represent standard deviations of technical replicates

Traditionally, the correlation between the biomass specific ATP production rate (q_ATP_) and the growth rate (μ) in chemostat cultures is used to determine the maximum biomass specific yield on ATP (Y_x/ATP_^max^) and the maintenance coefficient (m_ATP_). However, this approach only takes into account growth rates above approximately 0.05 h^−1^ and therefore deviations at low growth rates cannot be found. Based on dynamic modelling of retentostat cultures, it was estimated that the m_ATP_ of *L. lactis* FM03-V1 decreases sevenfold at near-zero growth rates [8]. We used the biomass concentrations and the metabolite production rates in the steady states of the PCRC culture to calculate q_ATP_, which was plotted against the growth rate (Fig. 2). We calculated the ATP production rate using Eq. 5.

5 $$R_{ATP} = R_{lactate} + R_{ethanol} + R_{pyruvate} + 2 \cdot R_{acetoin} + 2 \cdot R_{acetate}$$

in which R_i_ is the production rate of compound i.

The q_ATP_ was significantly lower at near-zero growth rates than estimates based on extrapolations of chemostat cultures (3.6-fold at 0.0025 h^−1^), confirming the decreased maintenance coefficient at near-zero growth rates. Moreover, the extent of this decrease seemed to increase towards lower growth rates. To further study the relation between the maintenance and the growth rate, the biomass accumulation in the PCRC was modelled assuming that the maintenance coefficient exponentially decreased at low growth rates (see Eq. 4). The obtained model excellently fitted the data (root mean squared error = 0.035 gDW/kg; Additional file 2: Figure S4) and is in line with the traditional model estimating the maximum biomass yield and maintenance coefficient using conventional chemostat cultures (Fig. 2). Extrapolations of the traditional model with a constant maintenance coefficient and the new model with an exponentially decreasing maintenance coefficient showed that the maintenance coefficient decreases approximately 6.4-fold when the growth rate decreases to zero.

#### Volatile organic compound production

**Fig. 3:**
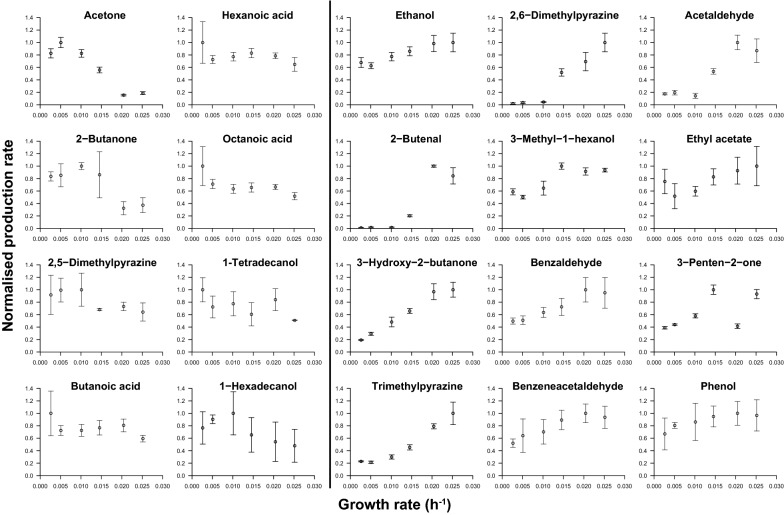
Aroma production by *L. lactis* FM03-V2 in a partial cell recycling chemostat culture at different growth rates. The eight compounds in the two columns on the left increased at lower growth rates, while the 12 compounds on the right decreased. The production rate is calculated based on the abundance in the cell-free permeate and cell-containing effluent and their corresponding flow rates and afterwards normalized per compound by dividing each rate by the highest production rate. Error bars represent the standard deviation of technical duplicates

To identify changes in the metabolism resulting from the low growth rates, volatile organic compounds were analyzed in the steady states by HS SPME GC–MS. Both complete and cell-free samples were analyzed to estimate the production rates because particular compounds are predominantly located in the cell membrane due to their hydrophobic nature [8]. In total 27 compounds were identified. Based on the flow rates of the effluent (corresponding to the complete samples) and the permeate (corresponding to cell-free samples) combined with the determined abundances of the metabolites in both samples, we calculated the production rates of each compound (Fig. 3). In general, the production of ketones, fatty acids and long-chain alcohols increased towards low growth rates (i.e. acetone, 2-butanone, butanoic acid, hexanoic acid and, octanoic acid, 1-tetradecanol and 1-hexadecanol), while aldehydes, acetoin and pyrazines decreased (i.e. acetaldehyde, benzaldehyde, benzeneacetaldehyde, 2-butenal, 3-hydroxy-2-butanone, trimethylpyrazine and 2,6-dimethylpyrazine). An increase of ketones and fatty acids at lower growth rates was also found for *L. lactis* FM03-V1 during retentostat cultivation [8].

### Discussion

Partial cell recycling chemostat (PCRC) cultivation is a promising technique because higher biomass and metabolites production rates can be obtained compared to batch and chemostat cultures [10, 11]. We demonstrated that this system could also be used to cultivate microorganisms in steady states at very low growth rates to continuously produce metabolites typical for slow growth and to study the microbial physiology.

By cultivation of *L. lactis* FM03-V2 at growth rates between 0.0025 and 0.025 h^−1^ in a PCRC, we demonstrated that the maintenance coefficient of this strain gradually decreases at near-zero growth rates compared to high growth rate (> 0.05 h^−1^), which corresponds well with previous predictions using dynamic modelling of retentostat cultures (sevenfold) [8]. The high viability (> 99%) during the cultivation indicates that this reduction was not caused by an inactive or dead fraction of cells. An exponentially decreasing maintenance coefficient was included in a dynamic growth model, which allowed accurate prediction of the biomass accumulation in the PCRC culture.

During the cultivation, *L. lactis* converted lactose mainly into lactate, acetate, ethanol, formate and pyruvate. It is known that *L. lactis* shifts from homolactic fermentation to mixed-acid fermentation (combination of acetate, formate and ethanol) at low growth rates [12, 13] as was found for *L. lactis* FM03-V1 (Additional file 2: Figure S5). Surprisingly, we found an increase in lactic acid production at near-zero growth rates, which cannot be explained by the trade-off hypothesis explaining the shift as trade-off between the metabolic and energetic efficiency of the pathways and the cost of synthesizing the enzymes [14].

In general, levels of acids and ketones increased at lower growth rates, while aldehydes, acetoin and pyrazines decreased. Ketones and acids are groups that contain important aroma compounds in fermented dairy products, like cheese [15]. Production of most of these compounds is related to the lipolytic activity of moulds, but our study shows that they can also be produced by *L. lactis* FM03-V2 at extremely low growth rates.

This study demonstrates that PCRC cultivation can be used to study microorganisms at extremely low growth rates in static conditions and has important advantages over retentostat cultivation. The culture viability was higher during prolonged cultivation in PCRCs (> 99% instead of ~ 80%). Moreover, the PCRC is likely to be more reproducible due to its time-independency and less affected by small disturbances by sampling, clogging of the filter or changes in the feed rate. This makes the PCRC more suitable for omics studies, such as transcriptomics, proteomics and metabolomics. Furthermore, it offers the unique opportunity to study the impact of cell density and quorum sensing-dependent behavior at a constant growth rate under constant environmental conditions by only changing the feed rate and the recycle ratio.

In conclusion, PCRC cultivation is a unique cultivation technique that has both promising applications in biotechnological research, food production processes and in academic research.

## Limitations

This study contains only one biological replicate of the partial cell recycling chemostat cultivation and therefore the reproducibility of this technique has to be confirmed.

## Additional files

**Additional file 1.** Derivation of equation 1 describing biomass accumulation in partial cell recycling chemostat cultures.

**Additional file 2: Figure S1.** Schematic overview of the partial cell recycling chemostat set-up. **Figure S2**: Relation between the online optical density and the biomass concentration. **Figure S3**: Metabolite production by *L. lactis* FM03-V2 in a partial cell recycling chemostat culture as function of the growth rate. **Figure S4**: Prediction of the biomass concentration in the partial cell recycling chemostat culture. **Figure S5**: Metabolite production by *L. lactis* FM03-V1 in a chemostat culture as function of the growth rate.

## Abbreviations

µ: specific growth rate (h^−1^)
C_s_: lactose concentration in the reactor
C_s,in_: lactose concentration in the medium
C_x_: biomass concentration in the bioreactor (gDW kg^−1^)
F: flow of medium (L h^−1^)
HS SPME GCMS: headspace solid phase microextraction gas chromatography mass spectrometry
HPLC: high performance liquid chromatography
m_ATP_: energy-related maintenance coefficient (mol ATP gDW^−1^ h^−1^)
m_ATP,che_: energy-related maintenance coefficient estimated in chemostat cultures (mol ATP gDW^−1^ h^−1^)
PCRC: partial cell recycling chemostat
PDH: pyruvate dehydrogenase
PFL: pyruvate formate lyase
q_ATP_: biomass specific ATP production rate (mol ATP gDW^−1^ h^−1^)
R: recycle ratio
R_i_: production rate of compound i (mol h^−1^)
t: time (h)
V: volume (L)
Y_ATP/s_: ATP yield on substrate (mol ATP/CmolS)
Y_x/ATP_^max^: maximum biomass yield on ATP (gDW mol^−1^ ATP)
