# Supplementary material for: Application of a partial cell recycling chemostat for continuous production of aroma compounds at near-zero growth rates
Source: BMC Res Notes. 2019 Mar 25;12:173. doi: 10.1186/s13104-019-4213-4 (PMC6434626; doi:10.1186/s13104-019-4213-4)
Supplement: Supplementary file 1 — Additional file 1. Derivation of equation 1 describing biomass accumulation in partial cell recycling chemostat cultures. [file 13104_2019_4213_MOESM1_ESM.pdf]

Additional File 1: Derivation of Equation 1 describing biomass accumulation in partial cell recycling chemostat cultures

To estimate the maintenance coefficient during the partial cell recycling chemostat cultivation, biomass accumulation was modelled based on: i) mass balances of biomass and substrate (Eq. 1.1 and 1.2), ii) the Herbert-Pirt equation (Eq. 1.3) describing how substrate is divided over growth and maintenance, and iii) changes in metabolism resulting in different ATP yield on substrate (Eq. 1.4 and 1.5). In these equations, C_x denotes biomass concentration in the reactor (gDW/kg), μ is the growth rate (h^{-1}), F is the flow of medium (L/h), V is the volume (L), R is the recycle ratio, $C_{s,out}$ is the substrate concentration in the reactor (no residual lactose), $C_{s,in}$ is the substrate concentration in the medium (0.1752 Cmol/L lactose), q_s is the biomass specific substrate consumption rate ($Cmol.gDW^{-1}.h^{-1}$), t is the time (h), $Y_{x/s}^{max}$ is the maximum biomass yield on substrate (gDW/CmolS), $Y_{x/ATP}^{max}$ is the maximum biomass yield on ATP (gDW/mol ATP), m_s is the substrate-related maintenance coefficient ($CmolS.gDW^{-1}.h^{-1}$), m_{ATP} is the energy-related maintenance coefficient (mol $ATP.gDW^{-1}.h^{-1}$) and $Y_{ATP/s}$ is the ATP yield on substrate (mol ATP/CmolS).

$$\frac{dC_x}{dt} = \mu \cdot C_x - \frac{F}{V}(1-R) \cdot C_x \qquad (1.1)$$

$$\frac{dC_s}{dt} = \frac{F}{V}\left(C_{s,in} - C_s\right) - q_s \cdot C_x \qquad (1.2)$$

$$q_s = \frac{\mu}{Y_{x/s}^{max}} + m_s \qquad (1.3)$$

$$Y_{x/s}^{max} = Y_{x/ATP}^{max} \cdot Y_{ATP/s} \qquad (1.4)$$

$$m_s = \frac{m_{ATP}}{Y_{ATP/s}} \qquad (1.5)$$

Because the lactose concentration in the bioreactor was much lower than the concentration in the medium and citrate was not consumed at all, a pseudo steady state can be assumed, i.e. $dC_s/dt = 0$. After substitution of equation 1.3 in equation 1.2, an expression for μ can be obtained, which can be substituted in equation 1.1. Integration of this equation and substitution of $Y_{x/s}^{max}$ and m_s using equation 1.4 and 1.5 leads to equation 1.6:

$$C_X(t) = \left(C_{x,0} - \frac{\left(\frac{F}{V}\cdot(C_{s,in}-C_s)\cdot Y_{x/ATP}^{max}\cdot Y_{ATP/s}\right)}{Y_{x/ATP}^{max}\cdot m_{ATP}-(1-R)\cdot\frac{F}{V}}\right)\cdot e^{-\left(Y_{x/ATP}^{max}\cdot m_{ATP}+\frac{F}{V}\cdot(1-R)\right)\cdot t} + \frac{\left(\frac{F}{V}\cdot(C_{s,in}-C_s)\cdot Y_{x/ATP}^{max}\cdot Y_{ATP/s}\right)}{Y_{x/ATP}^{max}\cdot m_{ATP}-(1-R)\cdot\frac{F}{V}} \qquad (1.6)$$
