# Supplementary material for: Application of a partial cell recycling chemostat for continuous production of aroma compounds at near-zero growth rates
Source: BMC Res Notes. 2019 Mar 25;12:173. doi: 10.1186/s13104-019-4213-4 (PMC6434626; doi:10.1186/s13104-019-4213-4)
Supplement: Supplementary file 2 — Additional file 2: Figure S1. Schematic overview of the partial cell recycling chemostat set-up. Figure S2: Relation between the online optical density and the biomass concentration. Figure S3: Metabolite production by L. lactis FM03-V2 in a partial cell recycling chemostat culture as function of the growth rate. Figure S4: Prediction of the biomass concentration in the partial cell recycling chemostat culture. Figure S5: Metabolite production by L. lactis FM03-V1 in a chemostat culture as function of the growth rate. [file 13104_2019_4213_MOESM2_ESM.pdf]

Additional File 2

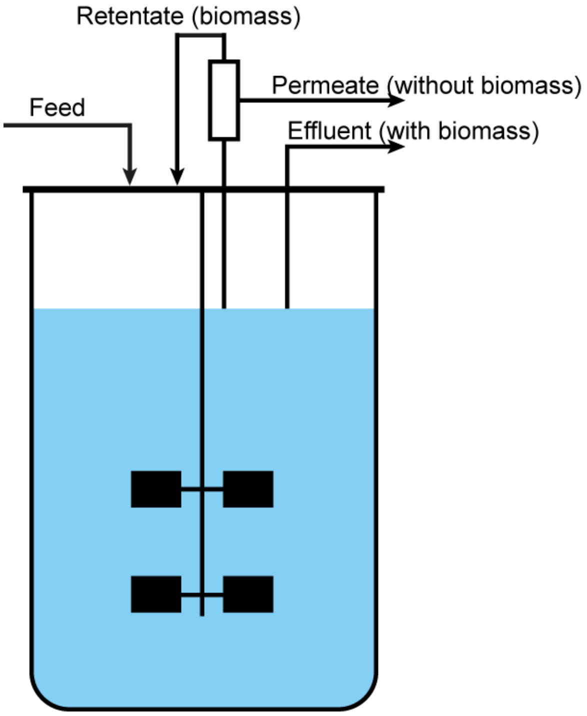

Fig. S1: Schematic overview of the partial cell recycling chemostat set-up.

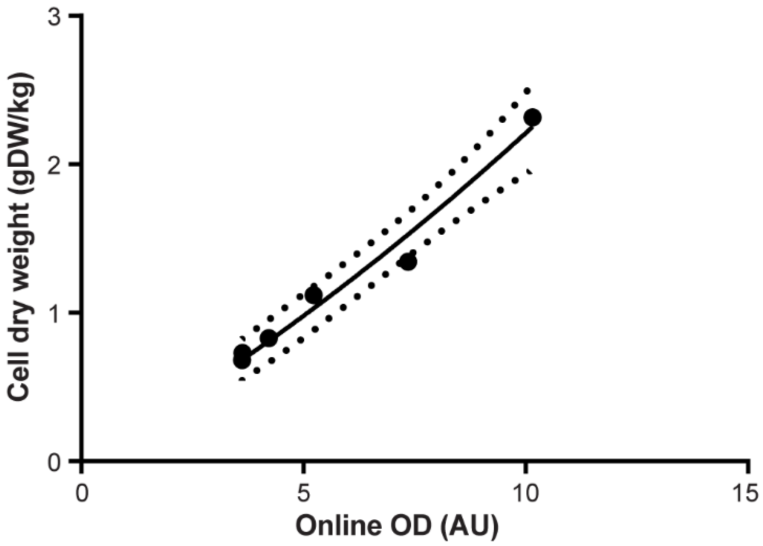

Fig. S2: Relation between the optical density at 600 nm, measured continuously with an internal probe, and the biomass concentration. The solid line represents a second-order polynomial function and the dotted lines represent the 95% confidence interval.

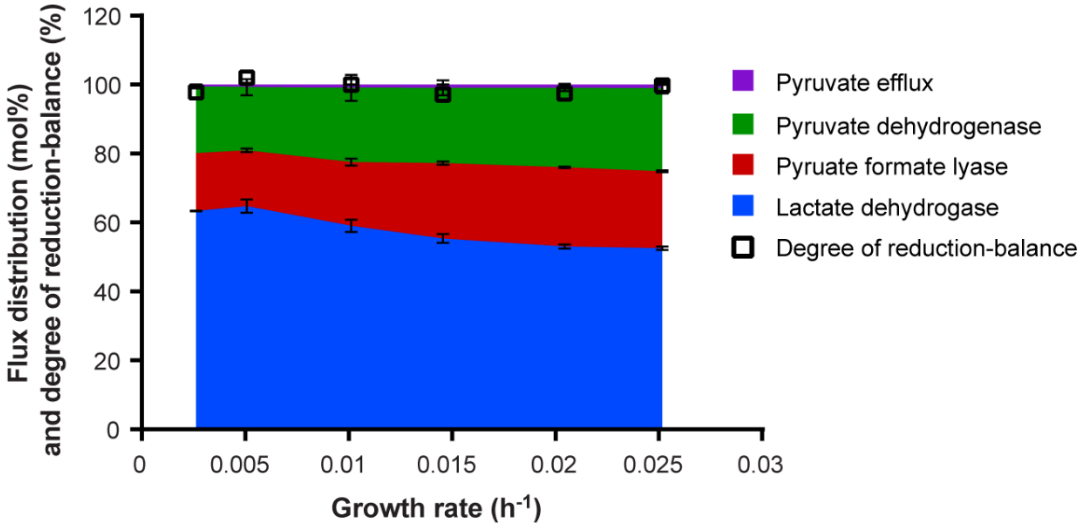

Fig. S3: Metabolite production by *L. lactis* FM03-V2 in a partial cell recycling chemostat culture as function of the growth rate. Metabolite concentrations of lactate, acetate, ethanol, formate and pyruvate were converted to fluxes of metabolic reactions consuming pyruvate and the four different fluxes were normalized to 100%. Error bars represent the standard deviation of technical duplicates.

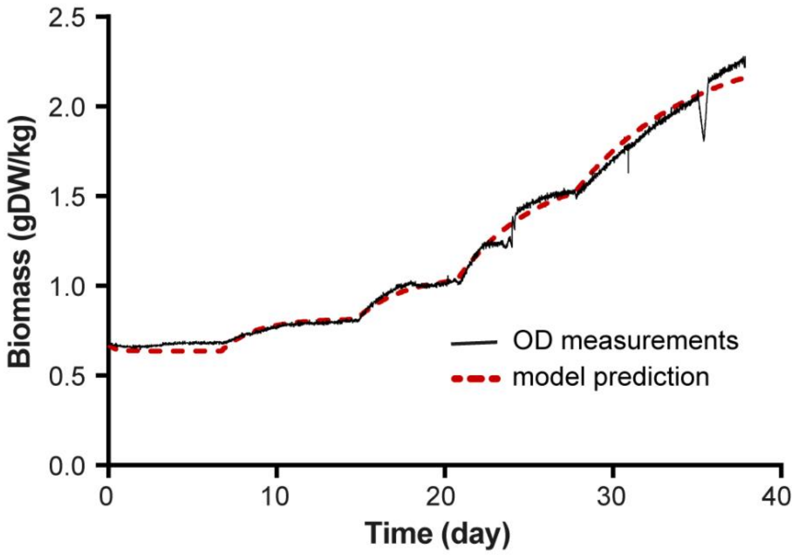

Fig. S4: Prediction of the biomass concentration in the partial cell recycling chemostat culture based on a exponentially decreasing maintenance coefficient towards lower growth rates (red dashed line). The black line represents the optical density measurements at 600 nm, which were converted to dry weight concentration with a second-order polynomial function (Fig. S2).

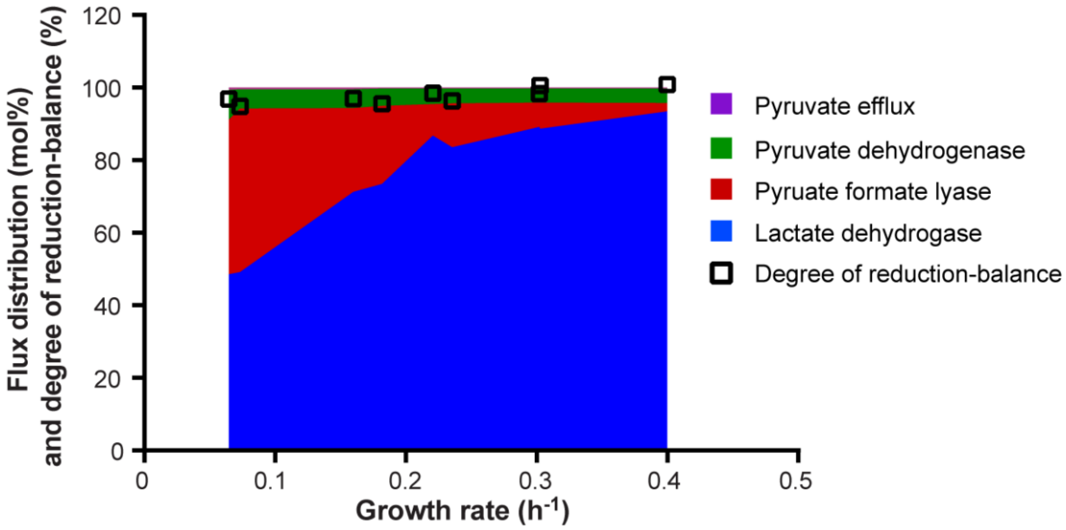

Fig. S5: Metabolite production by *L. lactis* FM03-V1 in a chemostat culture as function of the growth rate. Metabolite concentrations of lactate, acetate, ethanol, formate and pyruvate were converted to fluxes of metabolic reactions consuming pyruvate and the four different fluxes were normalized to 100%.
